# Infrastructure of tobacco and nicotine cessation services in Finnish healthcare: A cross-sectional study

**DOI:** 10.18332/tid/219129

**Published:** 2026-05-26

**Authors:** Tuija Ylitörmänen, Teija Strand, Hanna Ollila

**Affiliations:** 1 Department of Healthcare and Social Welfare, Finnish Institute for Health and Welfare, Helsinki, Finland

**Keywords:** tobacco cessation, early identification, brief intervention, infrastructure, healthcare service, JA-PreventNCD

## Abstract

**INTRODUCTION:**

Many countries have made significant progress in implementing effective tobacco control policies. Despite the availability of clinical treatment guidelines for smoking cessation, their integration into routine healthcare systems is less well-known. In Finland, recommendations addressing the infrastructure of tobacco and nicotine cessation, aimed at healthcare service providers, were published in 2024. This study aims to provide a baseline assessment for their evaluation.

**METHODS:**

This cross-sectional study collected data in autumn 2024 from twenty-one wellbeing service counties (88%) using an online survey directed at professionals working in healthcare services involved in tobacco and nicotine-related care, including smoking cessation counseling, early identification, and brief interventions. Data were analyzed using descriptive statistics. HHS Framework to Support and Accelerate Smoking Cessation 2024 was utilized to provide a conceptual framework for interpreting the findings.

**RESULTS:**

Established structures for cessation services were limited; only 29% (6/21) of counties had appointed a dedicated entity, and 33% (7/21) provided annual training for healthcare professionals in tobacco cessation. Documentation of tobacco and nicotine use in medical records was the most successfully implemented component, reported by 81% (17/21) of counties, followed by the provision of digital services, 71% (15/21). The system includes features for structural documentation, with 62% (13/21) of counties recording early identification and brief interventions in medical records. Cessation services were communicated through multiple channels, including websites, social media, and healthcare centers.

**CONCLUSIONS:**

There is a significant variation in the structure and organization of tobacco and nicotine cessation services across wellbeing service counties, with many still in the early stages of development. While documentation of tobacco and nicotine use among patients has been increasingly adopted, the implementation of key components such as early identification, brief interventions, and cessation support remains underemphasized. Strengthening institutional support, organizational structures, professional training, communication, and information flow are associated with more effective implementation.

## INTRODUCTION

Smoking is one of the greatest threats to public health and the leading preventable cause of premature death^1^. The use of tobacco and nicotine products hinders the prevention and treatment of key chronic diseases and slows down healing and recovery from diseases^1,2^. Even though tobacco and nicotine use have declined in many countries, smoking rates continue to be high^3^. The use of new nicotine products, especially e-cigarettes, has become more common^4^. As efforts to control tobacco use grow, it is crucial to expand comprehensive cessation services to help current tobacco users quit. The most significant impact of tobacco-prevention efforts is achieved when initiatives are implemented in coordination at both national and local levels, leveraging national policy frameworks alongside community-based interventions^5^.

Article 14 of the WHO Framework Convention on Tobacco Control (WHO FCTC) obligates Parties to implement measures that support tobacco and nicotine cessation and treat tobacco dependence. Despite the obligation, only about half of the Parties recognized measures under WHO Article 14 as a national priority in 2023. Implementation gaps, such as a lack of a national quit line, promotion of tobacco cessation services, and absence of cessation services in primary healthcare, were mentioned by the Parties^6^. A qualitative study assessed the implementation of WHO Article 14 in 127 countries, and found, among other things, a lack of a healthcare system infrastructure, low political priority, and lack of funding, as well as barriers to implementing the recommendations^7^. Another study examined the progress of the implementation of the WHO FCTC Article 14 and its guidelines in 142 countries. The results revealed that the implementation of the recommendation is slow. Overall, only 54% of countries had a designated person for tobacco dependence treatment, while fewer had guidelines (40%), a national strategy (32%), specialized services (26%), a budget (25%), quitlines (23%), or text messaging (17%)^8^.

In line with this, the World Health Organization (WHO) released a clinical treatment guideline for tobacco cessation in July 2024, recommending system-level strategies to embed cessation into healthcare systems. Cessation infrastructure refers to the system-level structures, policies, resources, and organizational support within the health system that enable the routine delivery of evidence-based tobacco cessation interventions. These strategies include routine screening for tobacco use, integrating cessation services into primary care, training health workers, ensuring access to evidence-based treatments, expanding digital tools and guidelines, and establishing monitoring systems to track outcomes. These recommendations are designed to support stakeholders in building sustainable, equitable, and effective cessation infrastructures^9^.

Similarly, in 2023, the U.S. Department of Health and Human Services (HHS) introduced a framework to accelerate smoking cessation and reduce health disparities by leveraging existing activities and collaborations. The vision is to ensure that everyone has access to comprehensive, evidence-based cessation treatments and supportive programs^10^.

Finland has also published and regularly updated clinical guidelines on tobacco and nicotine cessation methods since 2002. In 2023, a ministerial working group proposed – among other actions to support Finland’s goal of less than 5% of tobacco and nicotine use by the year 2030 – that the Ministry of Social Affairs and Health and the Finnish Institute for Health and Welfare prepare joint recommendations on tobacco and nicotine cessation to the wellbeing service counties, which are the healthcare providers in Finland^11^. These recommendations were published in 2024, with emphasis on the systematic organization and implementation of cessation services^12^. The recommendations are divided into six subscales: structures, knowledge management, strengthening of competence, communication, good practices, and people with special support needs. Key elements include clear coordination roles, defined care pathways, structured documentation, regular staff training, effective communication strategies, standardized operating models, and tailored approaches for those requiring additional support.

Further, under Finnish law, wellbeing service counties are required to prepare a welfare report as part of their statutory duties. This report provides an overview of the population’s health and wellbeing and outlines measures to promote them. The welfare report must be prepared once per parliamentary term (every four years) as an extensive report, and its objectives and measures are updated annually as part of planning and monitoring work^13^. Incorporating the aims and related actions of tobacco and nicotine cessation recommendations into the mandatory welfare report facilitates their systematic evaluation, monitoring, and follow-up.

Supporting individuals in quitting the use of tobacco and nicotine products, as well as preventing initiation, can enhance their health and quality of life^14^. Moreover, this can lead to substantial cost savings in both healthcare and society^5^. Nevertheless, smoking cessation guidelines and treatment pathways are not being effectively integrated into routine healthcare practices^15,16^. This might hinder clients’ access to tobacco cessation support and services. Barriers such as limited time, low confidence in discussing cessation, unclear roles for primary care, lack of tools to identify smokers, and insufficient performance incentives hinder effective tobacco treatment delivery in primary care^2^.

Structured documentation of tobacco use, snus, and other nicotine products, as well as cessation activities, is essential. Structured data consist of clearly defined and organized information that is recorded in the patient information system in accordance with standardized documentation practices. It enables the planning, monitoring, and evaluation of service delivery and effectiveness. Furthermore, it supports the assessment of the quality of care for public health diseases at both local and national levels, promoting consistent and evidence-based treatment practices^12^. The current extent and consistency of the infrastructure across wellbeing service counties have not been systematically examined. This study aims to establish a baseline of existing infrastructures for the tobacco cessation services. It addresses two main research questions: what the current state of tobacco and nicotine cessation service infrastructure is in the wellbeing service counties, and what resources and capacities are available to support the delivery of cessation services.

## METHODS

### Study design

This study employed a cross-sectional, descriptive, and quantitative design as part of a pilot implemented under the Joint Action Prevent Non-Communicable Diseases (JAPreventNCD) project. It is an EU initiative that supports countries in strengthening policies and practices to reduce the burden of cancer and other non-communicable diseases. The project focuses on improving prevention, early detection, and coordinated actions that address both individual and societal risk factors^17^. Finland’s health and social care system is publicly funded and organized into wellbeing services counties, which provide comprehensive healthcare, including primary care, specialized care, and preventive services. Tobacco and nicotine cessation support is integrated into this structure. Cessation services are offered through primary care, hospitals, and digital platforms, complemented by quitlines and counseling^18^.

### Setting and participants

The survey, administered electronically, was directed at professionals working in healthcare services involved in tobacco and nicotine-related care, including smoking cessation counseling, early identification, and brief interventions. Only one response was requested from each wellbeing services county. However, it was strongly encouraged that the response reflects the perspectives of experts working across various services. The survey was distributed to all 21 wellbeing services counties in Finland, as well as to the City of Helsinki, although not part of a wellbeing services county, which is responsible for organizing health, social, and rescue services. Additionally, the survey was sent to the HUS Group, which provides highly specialized healthcare, and to the autonomous region of Åland. In total, 24 entities were invited to participate. An information notice describing the project and the associated study, along with details of the existing ethical approval, was submitted to the registries of the wellbeing service counties. The registries forwarded the survey either directly to the intended respondents or to the persons responsible for research within their organizations. The response time for the survey was three weeks, after which two reminders were sent, extending the response time by one week at a time.

### Measures

The questionnaire was developed based on the Finnish Recommendations to the wellbeing services counties on smoking and nicotine cessation services^12^. It comprises 27 questions with sub-questions measuring six subscales (Supplementary file Table 1). Internal consistency for the subscales ranged from poor to good. The structures subscale (eight items) showed acceptable reliability (α=0.75), knowledge management (15 items) showed good reliability (α=0.88), and strengthening of competence (four items) showed questionable reliability (α=0.64). The communications subscale (nine items) demonstrated poor reliability (α=0.53), good practices (ten items) showed acceptable reliability (α=0.73), and people with special support needs (12 items) demonstrated good reliability (α=0.89). Participants responded to the survey items using a categorical scale comprising four options: ‘Yes’, ‘No, but planned’, ‘No’, and ‘I don’t know’. Additionally, the survey included questions involving background variables, such as the name of the wellbeing services county, range of responsibility, operating unit, position, other experts’ participation in answering the survey, and if the wellbeing services county is a member of the Finnish Network of Health Promoting Hospitals and Health Services (results not shown due to small data). The questionnaire was built and modified based on the Global Network for Tobacco-free Healthcare Services (GNTH) questionnaire^19^, which includes eight standards: governance and commitment, communication, education and training, identification, diagnosis and tobacco cessation support, tobacco-free environment, healthy workplace, community engagement, and monitoring and evaluation.

The U.S. Framework to Support and Accelerate Smoking Cessation 2024^10^ was employed to guide cessation efforts. The framework’s goals include reducing disparities, increasing knowledge, strengthening and sustaining cessation services, expanding access to treatment, enhancing surveillance and evaluation, and promoting research. These goals are consistent with the Finnish tobacco and nicotine cessation guidelines and recommendations^12^, thereby supporting their applicability within the Finnish healthcare context. The results section is structured around this framework, providing a theoretically grounded perspective for interpreting the findings.

Given that Finland has two official languages, the questionnaire was translated into Swedish and subsequently reviewed by a native Swedish speaker to ensure linguistic accuracy. The questionnaire was commented on by the Finnish national network for health-promoting hospitals and pre-tested by two experts with substantial knowledge in the field. Based on their feedback, minor wording adjustments were made.

### Data analysis

The data were analyzed with the Statistical Package for Social Sciences (SPSS for Windows 29.0, IBM, Armonk, NY)^20^. Descriptive statistics were calculated, including frequencies and percentage distributions. The internal consistency of the scale was assessed through Cronbach’s alpha. As no missing data were present, all analyses were conducted using the full data set.

### Ethical approval and consent

Ethical approval for the study’s design and conduct was obtained from the Institutional Review Board (IRB) of the Finnish Institute for Health and Welfare (THL/2531/6.02.01/2024). The study’s purpose, a note about its voluntary nature, and a confidentiality pledge were attached to the questionnaire. Completion of the questionnaire was taken as consent to participation. No wellbeing service county requested a separate research permit application.

## RESULTS

A total of 21 out of 24 wellbeing service counties responded to the survey. The number 24 includes Åland, the City of Helsinki, and Helsinki University Hospital (HUS), which differ administratively from wellbeing service counties but were counted due to their partial responsibility for healthcare and social services.

### Strengthen, expand, and sustain cessation services and supports

Approximately one third (29%) of the well-being services counties had an established entity and designated person(s) responsible for coordinating tobacco and nicotine cessation efforts, including the implementation of a common treatment path for smoking and nicotine cessation. Two counties (10%) had established a unit to provide the service. In nine (43%) wellbeing services counties, a preliminary agreement had been reached on a regional common practice for monitoring tobacco and nicotine cessation activities. In eight (38%) counties, a preliminary agreement had also been made regarding a shared regional approach to evaluating the outcomes of cessation efforts.

### Advance, expand, and sustain surveillance and strengthen performance measurement and evaluation

**Figure 1:**
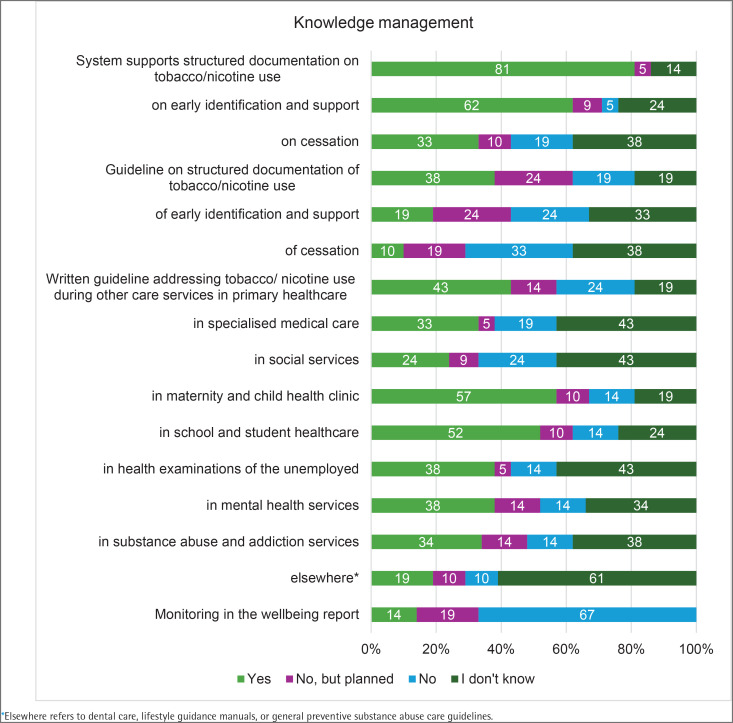
Knowledge management (% of respondents) of tobacco and nicotine cessation practices across wellbeing service counties, HUS Joint Authority, and the City of Helsinki, Finland, 2024 (N=21)

The patient and client information system enabled structured documentation of residents’ tobacco and nicotine use in 17 (81%) wellbeing service counties, as well as recording early identification and support measures, such as brief interventions, in 13 (62%) counties. Individual or group-based cessation counseling was available in seven (33%) counties. Additionally, eight (38%) counties reported having unit-level or system-level guidelines related to structured documentation of tobacco and nicotine use. Four (19%) counties had guidelines covering early identification and support, and two (10%) had guidelines for cessation activities. In addition, two to five counties were planning similar guidelines. A common written guideline for social and healthcare professionals on addressing tobacco and nicotine use during the delivery of other support and care services was available in several wellbeing services counties, most notably in maternity and child health clinics (12 counties, 57%), school and student healthcare (11 counties, 52%), and primary healthcare (9 counties, 43%). Systematic monitoring, evaluation, and reporting of cessation outcomes were included in the wellbeing report in three (14%) of the wellbeing service counties (Figure 1).

### Increase access to and coverage of comprehensive, evidence-based cessation treatment

**Figure 2:**
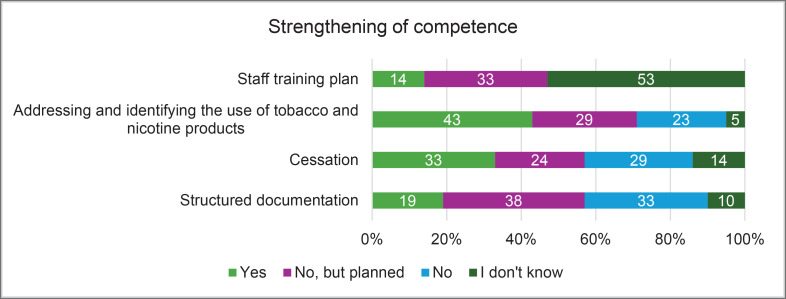
Strengthening competencies (% of respondents) in tobacco and nicotine cessation practices across wellbeing service counties, HUS Joint Authority, and the City of Helsinki, Finland, 2024 (N=21)

Three (14%) wellbeing services counties had a training plan for social and healthcare professionals on early identification and support related to tobacco and nicotine use, as well as cessation activities. Annual training for health and social care professionals on addressing and identifying tobacco and nicotine use was organized in some wellbeing service counties. Training on identifying the use of tobacco and nicotine products was provided in nine (43%) counties and planned in six (29%) additional counties. Tobacco cessation was included in training in seven (33%) counties, with five (24%) more planning to implement it. Structured documentation was part of training in four (19%) counties, and eight (38%) counties reported plans to include it in future training (Figure 2).

### Increase awareness and knowledge related to smoking and cessation

Communication practices related to tobacco and nicotine cessation varied across wellbeing service counties. In six (29%) counties, staff reported that it was easy to find information about their unit’s cessation model/procedure, while several others were planning improvements. Clear and appropriate materials to support client work were available in 14 (67%) counties. Information about cessation services was communicated through multiple channels, including websites (10 counties, 48%), social media (5 counties, 24%), healthcare centers (5 counties, 24%), and other formats (5 counties, 24%). Many counties used several of these methods in parallel. Only two (10%) wellbeing service counties reported having a jointly agreed practice for orienting new employees to tobacco and nicotine cessation procedures, while six (29%) counties indicated that such practices were being planned.

### Promote ongoing and innovative research to support and accelerate smoking cessation

Wellbeing services counties implement a range of best practices to support tobacco and nicotine cessation, both for clients and staff. Digital services were utilized as part of the service package in 15 (71%) counties, with attention to individual customer needs. Joint written instructions for drawing up a cessation plan together with the customer were available in four (19%) counties, and six (29%) counties had common instructions for offering nicotine replacement therapy to patients during ward treatment. Support for staff members who use tobacco and nicotine products was addressed in several ways. Individual counseling was available in eight (38%) counties, group tutoring in two (10%), nicotine replacement therapy in six (29%), and other drug treatment options in five (24%). Many counties applied multiple approaches simultaneously.

### Reduce smoking- and cessation-related disparities

**Figure 3:**
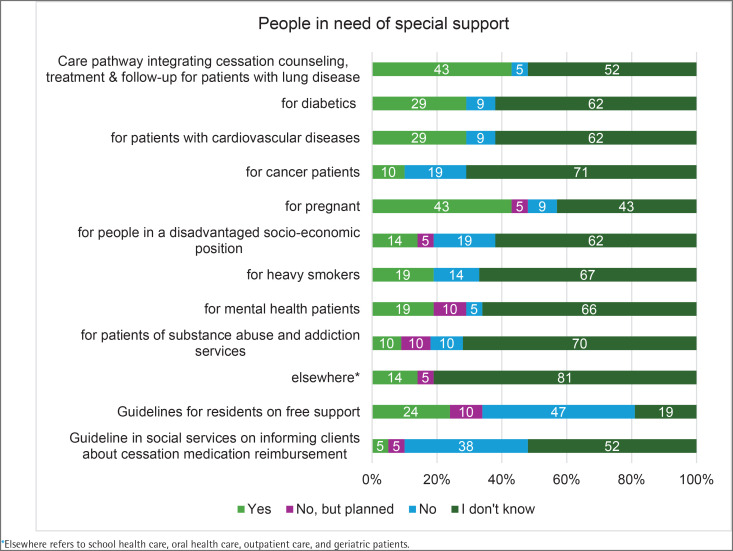
Implementation of tobacco and nicotine cessation practices (% of respondents) across wellbeing service counties, HUS Joint Authority, and the City of Helsinki, Finland, 2024 (N=21)

Service models or care pathways tailored for people in need of special support, where tobacco cessation counseling, treatment, and follow-up were integrated into other visits were reported in some wellbeing services counties. These include patients with lung diseases (9 counties, 43%), pregnant individuals (9 counties, 43%), diabetics (6 counties, 29%), and patients with cardiovascular diseases (6 counties, 29%). Fewer counties had tobacco cessation tailored for cancer patients (2 counties, 10%), people in disadvantaged socio-economic positions (3 counties, 14%), people who smoke heavily (4 counties, 19%), mental health service patients (4 counties, 19%), and patients with substance use disorders (2 counties, 10%). Joint written guidelines for offering free cessation support to residents existed in five (24%) counties. A guideline in social services on informing clients about reimbursement options for cessation medication was available only in one county (Figure 3).

## DISCUSSION

This baseline assessment of tobacco and nicotine cessation services across Finland’s wellbeing service counties, the HUS Joint Authority, the City of Helsinki, and Åland reveals heterogeneity in organization, implementation, and readiness. Key structural elements, such as the designation of a responsible entity, a responsible person for coordinating tobacco and nicotine cessation efforts, and care pathway descriptions, were only partially in place by autumn 2024. The designed person acts as a coordinator, expert, and support for new and other professionals, ensuring that efforts are strategic and impactful. The role of a designated person in tobacco and nicotine-related work, especially in providing annual training and cessation support, is considered important in the development, implementation, and long-term integration of these activities into everyday practices. Without the clear responsibility of a designed person, such work may become fragmented, inconsistent, or lose its effectiveness over time.

The observed gaps align with national recommendations^12^ and international guidance, which highlights the importance of clear governance structures, leadership support, and evidence-based models in organizing cessation services^21^. Implementation strategies, such as financial incentives (e.g. performance-based payments), infrastructure changes, and staff training, can enhance tobacco and nicotine cessation efforts^22^. Effective tobacco control efforts require broad, cross-sector collaboration to ensure the provision of comprehensive tobacco and nicotine cessation services and to reduce overall use^23^.

Processes such as systematic documentation of use, early identification, brief advice, and cessation actions varied widely, and shared practices for monitoring, reporting, and evaluation were often still under development. International models, such as the Ottawa Model for Smoking Cessation^24^, demonstrate that systematic recording and follow-up are important for effective cessation services. Tobacco surveillance provides essential information for strengthening tobacco control efforts, supporting the implementation of interventions, and assessing their impact^1^. The surveillance systems enable early detection, structured follow-up, and consistent documentation of cessation-related activities. Nationally defined data structures further support systematic documentation by providing information for situational awareness, monitoring progress, and guiding leadership decisions. Documented data increase the visibility of the work and enables timely allocation of resources.

Strengthening professional competence had begun in some counties but remained uneven. In certain services, written guidance and training were available; however, training content specific to cessation and education that supports structured documentation had not yet been implemented comprehensively. Systematic training has been shown to improve professionals’ ability to identify tobacco dependence, raise the topic proactively, and deliver evidence-based interventions^25^.

Communication plays a crucial role in increasing awareness and understanding of the health risks associated with tobacco and nicotine use. Communication practices across wellbeing service counties were fragmented. Some counties provided client materials and used multiple channels, yet the accessibility of information for staff and new employees remained insufficient. Effective internal communication ensures that personnel understand care pathways and can direct clients to services, while external communication increases public awareness and lowers barriers to seeking help^26,27^.

In recent years, counties have established effective new practices to support tobacco and nicotine-related work. Progress in digital cessation services is encouraging but incomplete. New digital services have been included in the service packages, indicating increased attention to innovative cessation approaches. The use of digital services not only expands access to services but also provides valuable data for assessing effectiveness. Earlier research has shown that integrated telemedicine predicts perceived autonomy support at three months, which in turn is linked to greater competence to quit and improved cessation outcomes^28^. However, guidance for developing individual cessation plans and implementing nicotine replacement therapy remained limited. WHO clinical guidance^9^ recommends combining digital interventions with behavioral counseling and pharmacotherapy to maximize effectiveness and reach.

Service models tailored to specific groups had been developed, but their coverage varied. Smoking and the use of nicotine products often accumulate among individuals who are, for example, facing socio-economic disadvantage or experiencing substance use and mental health-related challenges, which contribute significantly to health inequalities^29,30^. These groups often face greater challenges in quitting tobacco and nicotine use and are typically harder to engage in cessation programs^30^. Care pathways were most integrated for patients with pulmonary diseases and pregnant individuals, whereas services, for example, for people who smoke heavily, patients in mental health services, and those in lower socio-economic positions, were less common. Guidance on free cessation support was in place only in some counties. International reviews show that vulnerable groups share common barriers to quitting, such as using smoking to cope with stress, limited support from health and other service providers, and high acceptability of smoking within their communities^29^. Prior studies also indicate poorer service accessibility among more vulnerable groups^30,31^, suggesting that guidance for no-cost cessation support should be available in all counties to advance health equity. Implementation of strategies and measures to decrease tobacco and nicotine use among vulnerable groups is limited^32^ and a comprehensive approach is required to support smoking cessation in lower socio-economic groups^33^. Therefore, building models that demonstrate the value of cessation services can support advocacy efforts and help attract funding for a comprehensive approach^34^. These findings should be interpreted considering the limitations outlined in the strengths and limitations section.

### Strengths and limitations

This study provides useful information on the current organization of cessation services within Finnish healthcare settings. However, several limitations should be noted. Given that the study was conducted within Finland’s wellbeing services counties and the quantitative analysis was based on a small sample, the generalizability of the findings may be limited. Nevertheless, the findings offer meaningful insights given the limited existing research on organizational structures that support tobacco and nicotine cessation. A considerable proportion of ‘I don’t know’ responses suggests that cessation-related practices may still be under development or not yet systematically implemented across settings. These responses were retained, as excluding or merging them could have resulted in a loss of relevant information, and they may reflect gaps in knowledge or confidence among respondents.

Regarding the instrument, items were reviewed by experts to ensure content validity. Internal consistency varied across the six subscales, some showing only modest reliability, and therefore, conclusions based on these subscales should be interpreted with caution. Other subscales showed acceptable to good reliability, supporting the overall reliability of key components of the instrument. The cross-sectional design is another limitation, as it does not allow for causal interpretations. Although only one response per wellbeing services county was collected, respondents were encouraged to consult other professionals, and in some counties multiple contributors were involved. While this approach likely strengthened the accuracy of the responses, it may not fully capture the diversity of views within each county. As with all self-reported data, responses may also be influenced by social desirability bias.

## CONCLUSIONS

The systematic organization of tobacco and nicotine cessation services in Finland has begun, yet substantial heterogeneity in structures, processes, competence, and communication is producing inequities in access and quality. Following this baseline assessment, it will be important to establish ongoing mechanisms for monitoring and evaluating the implementation of the recommendations, while systematically examining the barriers and facilitators influencing this process. Future development should prioritize strengthening structural support and standardizing operating models across wellbeing service counties. Delivering effective and equitable cessation services depends on consistent implementation, professional capacity building, efficient communication, and a seamless flow of information. Enhancing these areas could support more consistent and high-quality delivery of early identification, brief advice, and cessation support.

## Supplementary Material



## Data Availability

The data supporting this research are available from the authors on a reasonable request.
