# Supplementary material for: Infrastructure of tobacco and nicotine cessation services in Finnish healthcare: A cross-sectional study
Source: Tob Induc Dis. 2026 May 26;24:10.18332/tid/219129. doi: 10.18332/tid/219129 (PMC13205645; doi:10.18332/tid/219129)
Supplement: Supplementary file 1 [file TID-24-71-s1.pdf]

Table 1. Early identification of tobacco and nicotine product use and the provision of cessation support. Cross-sectional survey among wellbeing services counties in Finland, 2024 (n = 21).

Subscales		Response options		
**Structures**	Yes	No, but planned	No	I don’t know
Does your wellbeing services county have a designated entity (e.g., a working group) responsible for coordinating tobacco and nicotine cessation?				
Does your wellbeing services county designate tobacco and nicotine cessation coordinators within its operational units/service areas who are responsible for developing the cessation operating model, coordinating its implementation, and overseeing its development and monitoring in collaboration with the county level- responsible body?				
Does your wellbeing services county have a separate unit dedicated to tobacco and nicotine cessation that provides cessation services to clients (e.g., a tobacco cessation clinic)?				
Does your wellbeing services county have a documented, shared care pathway for tobacco and nicotine cessation (e.g., municipal services – primary health care – specialized care – social services – occupational health services)?				
Has your wellbeing services county established a shared agreement on the monitoring, reporting, and evaluation of tobacco and nicotine cessation (e.g., as part of the preventive substance use plan)?<br>☐ Agreed on monitoring<br>☐ Agreed on reporting<br>☐ Agreed on evaluation				
Have annual targets been set for tobacco and nicotine cessation in your wellbeing services county?				
		Response options		
**Knowledge management**	Yes	No, but planned	No	I don’t know
Does your wellbeing services county’s patient/client information system enable the documentation of tobacco and nicotine product use, early identification and support, and cessation activities using the nationally defined data structures?<br>☐ Documentation of tobacco and nicotine product use<br>☐ Early identification and support<br>☐ Cessation activities				
Does your wellbeing services county have unit specific and information- system- specific- guidelines for the structured				

documentation of tobacco and nicotine product use, early identification and support, and cessation activities?

- ☐ Documentation of tobacco and nicotine product use
- ☐ Early identification and support
- ☐ Cessation activities

Has your wellbeing services county developed a shared written guideline for social and health care professionals on how to address tobacco and nicotine product use when providing other support and care across different services?

- ☐ in primary care
- ☐ specialised health care
- ☐ social services
- ☐ maternity and child health clinics
- ☐ school and student health services
- ☐ health services for the unemployed
- ☐ mental health services
- ☐ substance use and addiction services

Is the systematic monitoring, evaluation, and reporting of cessation outcomes included in your wellbeing services county's wellbeing report?

	Response options			
**Strengthening competence**	Yes	No, but planned	No	I don't know
Has your wellbeing services county developed a training plan / competence development plan for social and health care professionals related to the early identification and support of tobacco and nicotine product use, as well as cessation activities?				
Does your wellbeing services county provide annual training for social and health care staff on addressing and identifying tobacco and nicotine product use, on cessation, and on the structured documentation of use and cessation related-activities?<br>☐ identifying tobacco and nicotine product use<br>☐ on cessation<br>☐ on the structured documentation of use and cessation related- activities				

	Response options			
**Communication**	Yes	No, but planned	No	I don't know
Has a communication and advocacy plan been developed for internal and external communication related to tobacco and nicotine cessation?				
Has it been ensured that staff can easily find information				

about their unit’s tobacco and nicotine cessation operating model?				
Are clear and appropriate materials available to support client work related to tobacco and nicotine cessation?				
Has your wellbeing services county informed people who use tobacco and nicotine products about the available cessation services through various communication channels?<br>☐ the web pages<br>☐ social media<br>☐ health and social services centers<br>☐ other ways				
Has your wellbeing services county established a shared practice for onboarding new employees regarding the county’s tobacco and nicotine cessation procedures?<br>☐ about the operational model for smoking and nicotine cessation<br>☐ about the structured documentation of tobacco and nicotine product use and cessation				

**Good practices**	Yes	Response options<br>No, but planned	No	I don’t know
Does your wellbeing services county implement good practices in tobacco and nicotine cessation, for example through cooperation with networks and non-governmental organizations?				
Does your wellbeing services county utilize digital services as part of the tobacco and nicotine cessation service package, taking into account the client’s needs?				
Has your wellbeing services county developed a shared written guideline for creating a tobacco and nicotine cessation plan together with the client?				
Has your wellbeing services county developed a shared written guideline stating that tobacco and nicotine cessation counselling should be provided either individually or in group format?				
Has your wellbeing services county established a shared written guideline on providing nicotine replacement therapy during inpatient care for patients who use tobacco and nicotine products?				
Has your wellbeing services county established a shared written guideline on providing support to staff members who use tobacco and nicotine products?<br>☐ individual counselling<br>☐ group counselling				

- ☐ nicotine replacement therapy (NRT)
- ☐ other medication-based treatment

Has your wellbeing services county developed a shared written guideline on the provision of nicotine replacement therapy for staff and on how this is communicated?				

**Groups in need of special support**	Yes	Response options<br>No, but planned	No	I don't know
Does your wellbeing services county have an operating model or care pathway for the following groups, in which cessation counselling, treatment, and follow up- are integrated into other visits through an individualized cessation plan?<br>☐ patients with respiratory diseases<br>☐ patients with diabetes<br>☐ patients with cardiovascular diseases<br>☐ cancer patients<br>☐ pregnant women<br>☐ people with lower socioeconomic status<br>☐ people who smoke heavily<br>☐ people using mental health services				
Has your wellbeing services county established a shared written guideline on providing free support for residents to help them quit using tobacco and nicotine products?				
Has your wellbeing services county's social services established a shared written guideline on informing clients about the reimbursement options for cessation medications (e.g., health insurance, income support)?				

STROBE Statement—Checklist of items that should be included in reports of ***cross-sectional studies***

	Item No	Recommendation	Page No
**Title and abstract**	1	(*a*) Indicate the study's design with a commonly used term in the title or the abstract	1
		(*b*) Provide in the abstract an informative and balanced summary of what was done and what was found	1
**Introduction**			
Background/rationale	2	Explain the scientific background and rationale for the investigation being reported	2-3
Objectives	3	State specific objectives, including any prespecified hypotheses	3-4
**Methods**			
Study design	4	Present key elements of study design early in the paper	4
Setting	5	Describe the setting, locations, and relevant dates, including periods of recruitment, exposure, follow-up, and data collection	4
Participants	6	(*a*) Give the eligibility criteria, and the sources and methods of selection of participants	4
Variables	7	Clearly define all outcomes, exposures, predictors, potential confounders, and effect modifiers. Give diagnostic criteria, if applicable	4-5
Data sources/ measurement	8*	For each variable of interest, give sources of data and details of methods of assessment (measurement). Describe comparability of assessment methods if there is more than one group	*4-5*
Bias	9	Describe any efforts to address potential sources of bias	10
Study size	10	Explain how the study size was arrived at	4
Quantitative variables	11	Explain how quantitative variables were handled in the analyses. If applicable, describe which groupings were chosen and why	4-5
Statistical methods	12	(*a*) Describe all statistical methods, including those used to control for confounding	5
		(*b*) Describe any methods used to examine subgroups and interactions	5
		(*c*) Explain how missing data were addressed	
		(*d*) If applicable, describe analytical methods taking account of sampling strategy	5
		(*e*) Describe any sensitivity analyses	

**Results**			
Participants	13*	(a) Report numbers of individuals at each stage of study—eg numbers potentially eligible, examined for eligibility, confirmed eligible, included in the study, completing follow-up, and analysed	4
		(b) Give reasons for non-participation at each stage	
		(c) Consider use of a flow diagram	
Descriptive data	14*	(a) Give characteristics of study participants (eg demographic, clinical, social) and information on exposures and potential confounders	4
		(b) Indicate number of participants with missing data for each variable of interest	
Outcome data	15*	Report numbers of outcome events or summary measures	6-8, Fig 2-4
Main results	16	(*a*) Give unadjusted estimates and, if applicable, confounder-adjusted estimates and their precision (eg, 95% confidence interval). Make clear which confounders were adjusted for and why they were included	6-8
		(*b*) Report category boundaries when continuous variables were categorized	
		(*c*) If relevant, consider translating estimates of relative risk into absolute risk for a meaningful time period	
Other analyses	17	Report other analyses done—eg analyses of subgroups and interactions, and sensitivity analyses	
**Discussion**			
Key results	18	Summarise key results with reference to study objectives	8-10
Limitations	19	Discuss limitations of the study, taking into account sources of potential bias or imprecision. Discuss both direction and magnitude of any potential bias	10
Interpretation	20	Give a cautious overall interpretation of results considering objectives, limitations, multiplicity of analyses, results from similar studies, and other relevant evidence	8-10
Generalisability	21	Discuss the generalisability (external validity) of the study results	10
**Other information**			
Funding	22	Give the source of funding and the role of the funders for the present study and, if applicable, for the original study on which the present article is based	11

*Give information separately for exposed and unexposed groups.

Note: An Explanation and Elaboration article discusses each checklist item and gives methodological background and published examples of transparent reporting. The STROBE checklist is best used in conjunction with this article (freely available on the Web sites of PLoS Medicine at http://www.plosmedicine.org/, Annals of Internal Medicine at http://www.annals.org/, and Epidemiology at http://www.epidem.com/). Information on the STROBE Initiative is available at www.strobe-statement.org.

© 2026 Ylitörmänen T. et al.
